# Supplementary material for: Differences in the Sublethal Effects of Sulfoxaflor and Acetamiprid on the Aphis gossypii Glover (Homoptera: Aphididae) Are Related to Its Basic Sensitivity Level
Source: Insects. 2022 May 26;13(6):498. doi: 10.3390/insects13060498 (PMC9225309; doi:10.3390/insects13060498)
Supplement: Supplementary file 1 [file insects-13-00498-s001.zip › insects-1716911-supplementary.pdf]

Table S1. Statistical results of sublethal effects of acetamiprid on F_1 and F_2 generations

Stages	F_1 generation				F_2 generation			
	Jinghe		Yarkant		Jinghe		Yarkant	
	Control vs Acetamiprid		Control vs Acetamiprid		Control vs Acetamiprid		Control vs Acetamiprid	
	95% CI	*P*	95% CI	*P*	95% CI	*P*	95% CI	*P*
Pre-adult	(-0.08, 0.35)	0.240	(-5.01, 0.34)	0.153	(-0.10, 0.22)	0.481	(-1.52, 0.26)	0.086
Adult	(-1.56, 3.61)	0.436	(0.81, 4.09) *	0.003	(-0.32, 4.42)	0.093	(2.07, 4.92) *	0.000
APOP	(-0.01, 0.28)	0.061	(-0.14, 0.15)	0.950	(-0.11, 0.15)	0.769	(-0.13, 0.15)	0.901
TPOP	(0.03 0.48) *	0.026	(-0.10, 0.35)	0.267	(-0.13, 0.21)	0.645	(-0.01, 0.22)	0.082
Oviposition days	(-0.02, 2.47)	0.059	(-0.57, 1.61)	0.345	(-0.68, 1.54)	0.451	(1.45, 3.29) *	0.000
Total longevity	(-1.40, 3.71)	0.376	(0.70, 3.91) *	0.005	(-0.25, 4.46)	0.082	(1.95, 4.80) *	0.000
Fecundity	(-0.16, 10.25)	0.057	(1.30, 14.11) *	0.018	(-3.18, 6.99)	0.465	(15.51, 24.53) *	0.000
Parameters								
r	(0.01, 0.03)	0.244	(0.01, 0.06) *	0.002	(-0.01, 0.02)	0.750	(0.04, 0.08) *	0.000
λ	(-0.01, 0.04)	0.244	(0.02, 8.03) *	0.002	(-0.02, 0.03)	0.751	(0.06, 0.11) *	0.000
R_0	(-3.73, 8.29)	0.463	(2.44, 15.82) *	0.007	(-2.95, 9.24)	0.311	(17.82, 27.91) *	0.000
T	(0.19, 0.89) *	0.002	(-0.12, 0.43)	0.273	(-0.11, 0.33)	0.325	(-0.06, 0.42)	0.152
GRR	(-0.69, 7.68)	0.106	(-0.61, 10.29)	0.082	(-2.98, 4.51)	0.701	(9.06, 17.88) *	0.000

Paired Bootstrap test between two treatments was conducted by TWOSEX - MS Chart software. If 95% confidence interval contains 0, then there was no difference between the two treatments at the 5% level. An asterisk indicates a significant difference between the two treatments ($P < 0.05$).

Table S2. Statistical results of sublethal effects of sulfoxaflor on F_1 and F_2 generations

Stages	F_1 generation				F_2 generation			
	Jinghe		Yarkant		Jinghe		Yarkant	
	Control vs Sulfoxaflor		Control vs Sulfoxaflor		Control vs Sulfoxaflor		Control vs Sulfoxaflor	
	95% CI	*P*	95% CI	*P*	95% CI	*P*	95% CI	*P*
Pre-adult	(-0.14, 0.27)	0.547	(-0.14, 0.21)	0.706	(-0.13, 0.19)	0.720	(0.02, 0.29) *	0.024
Adult	(-1.68, 3.80)	0.449	(1.07, 4.66) *	0.002	(-0.72, 4.25)	0.160	(-1.14, 1.73)	0.676
APOP	(-0.08, 0.21)	0.392	(-0.10, 0.23)	0.430	(0.11, 0.13)	0.914	(-0.04, 0.22)	0.204
TPOP	(-0.10, 0.34)	0.284	(-0.12, 0.27)	0.437	(-0.12, 0.20)	0.657	(-0.05, 0.20)	0.269
Oviposition days	(0.44, 2.11)	0.194	(0.70, 3.91) *	0.005	(-0.52, 1.64)	0.307	(-0.07, 1.44)	0.078
Total longevity	(-1.59, 3.84)	0.417	(1.14, 4.67) *	0.001	(-0.66, 4.24)	0.149	(-1.29, 1.57)	0.839
Fecundity	(-1.11, 9.72)	0.120	(0.90, 13.15) *	0.024	(-5.03, 5.29)	0.972	(0.39, 9.22) *	0.032
Parameters								
r	(0.02, 0.02)	0.912	(-0.01, 0.03)	0.093	(0.01, 0.04) *	0.039	(0.01, 0.03) *	0.003
λ	(-0.03, 0.03)	0.911	(-0.01, 0.05)	0.093	(0.01, 0.07) *	0.039	(0.01, 0.05) *	0.003
R_0	(-4.44, 7.73)	0.598	(-2.03, 11.73)	0.165	(-4.40, 7.62)	0.612	(-0.86, 9.14)	0.103
T	(-0.27, 0.48)	0.577	(0.46, 0.93) *	0.000	(0.09, 0.57) *	0.009	(-0.01, 0.40)	0.055
GRR	(-1.49, 7.05)	0.205	(-0.89, 8.57)	0.120	(-0.41, 7.84)	0.081	(-0.75, 6.09)	0.116

Paired Bootstrap test between two treatments was conducted by TWOSEX - MS Chart software. If 95% confidence interval contains 0, then there was no difference between the two treatments at the 5% level. An asterisk indicates a significant difference between the two treatments ($P < 0.05$).
